# Air Pollution, Smoking, and Plasma Homocysteine

**DOI:** 10.1289/ehp.9517

**Published:** 2006-11-13

**Authors:** Andrea Baccarelli, Antonella Zanobetti, Ida Martinelli, Paolo Grillo, Lifang Hou, Guido Lanzani, Pier Mannuccio Mannucci, Pier Alberto Bertazzi, Joel Schwartz

**Affiliations:** 1 Department of Environmental Health, Harvard School of Public Health, Boston, Massachusetts, USA; 2 Department of Environmental and Occupational Health, IRCCS Maggiore Hospital, Mangiagalli and Regina Elena Foundation and University of Milan, Milan, Italy; 3 A. Bianchi Bonomi Haemophilia and Thrombosis Center, Department of Internal Medicine and Medical Specialties, University of Milan and IRCCS Maggiore Hospital, Mangiagalli and Regina Elena Foundation, Milan, Italy; 4 Occupational and Environmental Epidemiology Branch, Division of Cancer Epidemiology and Genetics, National Cancer Institute, National Institutes of Health, Department of Health and Human Services, Bethesda, Maryland, USA; 5 Air Quality Unit, Regional Environmental Protection Agency ARPA Lombardia, Milan, Italy

**Keywords:** air pollution, cardiovascular risk, generalized additive models, homocysteine, particulate matter, smoking

## Abstract

**Background:**

Mild hyperhomocysteinemia is independently associated with an increased risk of cardiovascular disease. Air pollution exposure induces short-term inflammatory changes that may determine hyperhomocysteinemia, particularly in the presence of a preexisting proinflammatory status such as that found in cigarette smokers.

**Objective:**

We examined the relation of air pollution levels with fasting and postmethionine-load total homocysteine (tHcy) in 1,213 normal subjects from Lombardia, Italy.

**Methods:**

We obtained hourly concentrations of particulate matter < 10 μm in aerodynamic diameter (PM_10_) and gaseous pollutants (carbon monoxide, nitrogen dioxide, sulfur dioxide_,_ ozone) from 53 monitoring sites covering the study area. We applied generalized additive models to compute standardized regression coefficients controlled for age, sex, body mass index, smoking, alcohol, hormone use, temperature, day of the year, and long-term trends.

**Results:**

The estimated difference in tHcy associated with an interquartile increase in average PM_10_ concentrations in the 24 hr before the study was nonsignificant [0.4%; 95% confidence interval (CI), −2.4 to 3.3 for fasting; and 1.1%, 95% CI, −1.5 to 3.7 for postmethionine-load tHcy]. In smokers, 24-hr PM_10_ levels were associated with 6.3% (95% CI, 1.3 to 11.6; *p* < 0.05) and 4.9% (95% CI, 0.5 to 9.6; *p* < 0.05) increases in fasting and postmethionine-load tHcy, respectively, but no association was seen in nonsmokers (*p*-interaction = 0.005 for fasting and 0.039 for postmethionine-load tHcy). Average 24-hr O_3_ concentrations were associated with significant differences in fasting tHcy (6.7%; 95% CI, 0.9 to 12.8; *p* < 0.05), but no consistent associations were found when postmethionine-load tHcy and/or 7-day average O_3_ concentrations were considered.

**Conclusions:**

Air particles may interact with cigarette smoking and increase plasma homocysteine in healthy subjects.

Several epidemiologic investigations have demonstrated that ambient air pollution is associated with increased risk of cardiovascular disease (Brook et al. 2004). In particular, acute increases in ambient particulate matter (PM) levels have been associated with myocardial infarction, stroke, and other adverse effects on cardiovascular function (Biggeri et al. 2004; Forastiere et al. 2005; Katsouyanni et al. 2001; O’Neill et al. 2005; Samet et al. 2000b; Schwartz et al. 2005; Wellenius et al. 2005; Zanobetti and Schwartz 2005; Zanobetti et al. 2004). The mechanisms linking inhalation of air pollutants to an increased cardiovascular risk are not fully understood (Bhatnagar 2006; Brook et al. 2002; Donaldson et al. 2001; Nel 2005; Peters et al. 1997, 2001).

Epidemiologic investigations have demonstrated that high plasma levels of total homocysteine (tHcy) are an independent risk factor for vascular disease, including coronary artery, cerebrovascular, and peripheral occlusive disease (Davey Smith and Ebrahim 2005; Welch and Loscalzo 1998). In addition to fasting tHcy measurements, tHcy determination after oral methionine load is used to identify individuals with mild impairment of Hcy metabolism, in whom fasting tHcy may be normal but postmethionine-load (PML) tHcy concentration is increased (Refsum et al. 2004). Alveolar and systemic inflammation has been proposed as a central component in the series of events linking the exposure to inhaled pollutants to the observed increases in cardiovascular morbidity and mortality (Brook et al. 2004; Seaton et al. 1995). Inflammation is a known determinant of hyperhomocysteinemia (Gori et al. 2005; von Eckardstein et al. 1994), and both air pollution and plasma tHcy have been associated with increased levels of C-reactive protein, fibrinogen, and interleukin-6 (Evans et al. 1997; Ghio et al. 2003; Gori et al. 2005; Pekkanen et al. 2000; Peters et al. 2001; Schwartz 2001; Seaton et al. 1999; von Eckardstein et al. 1994). However, whether air pollution exposure is correlated with increased tHcy levels has never been determined. Gori et al. (2005) suggested previously that short-term changes in inflammatory markers are associated with hyperhomocysteinemia when coupled with a mild persistent inflammatory state. In healthy subjects, tobacco smoking is a common cause of persistent low-level inflammation, and smoking has been also shown to induce elevated tHcy levels (Bazzano et al. 2003; De Bree et al. 2002; Guttormsen et al. 1996).

In the present study, we investigate the effects of air pollution levels on fasting and PML tHcy in 1,213 normal subjects from Lombardia, Italy. In addition, we examined potential effect modification by cigarette smoking of the relation between air pollution and tHcy.

## Materials and Methods

### Study population and laboratory methods

From January 1995 to August 2005, 1,218 healthy individuals, who were partners or friends of patients with thrombosis, attended the Thrombosis Center of the University of Milan, Italy, and agreed to undergo thrombophilia screening on a voluntary basis. Only individuals resident in the Lombardia region were chosen. Previous thrombosis was excluded with a validated structured questionnaire (Frezzato et al. 1996). None of the subjects was taking foline, vitamin B6, or vitamin B12 supplements. All participants gave written informed consent, and approval for the study was obtained from the University of Milan Departmental Institutional Review Board. On the day of the visit, the participants attended the Thrombosis Center at 0900 hr, when a first fasting blood sample was taken. A standardized questionnaire was administered including demographic data and questions on education, occupation, smoking, alcohol consumption, diet, reproductive history, and hormone use (oral contraceptives or hormone replacement therapy). Plasma tHcy was measured in EDTA anticoagulated blood samples, as previously described (Martinelli et al. 2003). Blood was withdrawn after overnight fasting for at least 8 hr, and again 4 hr after an oral methionine load (3.8 g/m^2^ body surface area). Blood samples were immediately placed on ice to prevent the artifactual *in vitro* increase in plasma tHcy levels and centrifuged at 1,600*g* at 4°C for 15 min within 1 hr. The supernatant platelet-poor plasma was stored at −80°C. Plasma tHcy was measured by high-performance liquid chromatography and fluorescence detection (Zighetti et al. 1997).

### Air pollution and weather data

**Figure 1:**
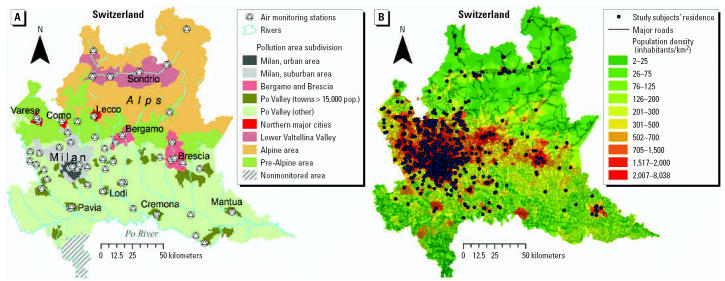
Lombardia region maps reporting (*A*) the location of the 53 air pollution monitors in the nine air pollution homogenous areas identified for the study, and (*B*) the residence of the study subjects.

We obtained from the Regional Environmental Protection Agency (ARPA Lombardia) recordings of hourly air pollution data measured from January 1994 to September 2005 by monitors located at 53 different sites throughout Lombardia (Figure 1A). The 53 stations included in this study were selected by ARPA Lombardia from the approximately 200 monitors of the Regional Air Monitoring Network on the basis of their location, reliability, determined by standardized quality control procedures and by correlation with *in situ* measurements, and continuity of recording. We identified nine different study areas in the region (Figure 1A) characterized by homogeneous within-area air pollution concentrations. Within each study area, levels of air pollutants measured by different monitors were highly correlated. The urban and suburban Milan areas (areas 1 and 2) included approximately 65% of the study subjects (Baccarelli et al. 2006) and had between-monitor correlations with *r* > 0.80 for all pollutants. In the remaining areas*,* between monitor correlations generally were > 0.70, with a few exceptions. In particular, correlations tended to be lower (*r* between 0.40–0.80) in area 3, which comprises two cities (Bergamo and Brescia) in a peculiar geographic location, partially enclosed in valleys at the Alps foothills. Although our analyses on pollution station data suggest that pollutant levels were quite heterogeneous in area 3, because only 18 subjects (1.5% of the total study population) were residents of this area, we did not modify the general strategy for exposure assignment for this area. In addition, mobile monitoring in each of the study areas during the study period showed high concordance with measurements taken by the permanent monitors in the same area (ARPA Lombardia 2006). For each study area, we averaged mean hourly concentrations of PM with an aerodynamic diameter ≤ 10 μm (PM_10_), carbon monoxide, nitrogen dioxide, sulphur dioxide, and ozone using an algorithm that combined levels reported by multiple monitoring locations (Schwartz 2000). We used these average concentrations for exposure assessment, after assigning each of the study subjects to one of the nine pollution areas, based on the subjects’ residence (Figure 1B). The southern part of the Pavia province (Figure 1A) was excluded, because this area had no local monitoring stations and showed pollution patterns in repeated point mobile recordings that differed from those measured by stationary monitors located in neighboring areas. Most air pollution stations also obtained data on weather, including air temperature, relative humidity, barometric pressure, intensity, and wind direction. We used data from the nearest Regional Weather Service surface station of the ARPA Lombardia network for stations that did not measure metereologic variables. In addition, we obtained data on mean daily linear visibility recorded at the three major airports (Milano Malpensa, Milano Linate, Bergamo Orio al Serio), and at one meteorologic station (Brescia-Ghedi) available online from the U.S. National Climatic Data Center (2006).

We used linear visibility data to calculate the extinction coefficient, which was shown to be a good predictor of fine particle concentrations (Ozkaynak et al. 1985). In most of the areas, total suspended particles (TSPs) rather than PM_10_ were measured in the earlier years of the study period (1995 in area 4; 1995–1996 in area 3; 1995–1997 in areas 1 and 2; and 1995–1998 in areas 5, 6, 7, and 9). TSP measurements were continued in the study areas after PM_10_ recording was introduced. For the periods in which only TSP measurements were available, we estimated PM_10_ as the predicted value from a model that included PM_10_ as the dependent variable and, as independent variables, day of the week, wind direction and penalized splines of TSP, temperature, barometric pressure, relative humidity, wind intensity, extinction coefficient, hour of the day, and date. The penalized splines were used to allow for nonlinear associations with PM_10_ concentrations. The analyses performed throughout this study were done including the predicted data. When predicted data were excluded from the analyses, the point estimates obtained were similar to those including predicted data, but had wider confidence intervals (CIs).

### Statistical analysis

In the analysis of the association of air pollutants with tHcy, the following variables were chosen *a priori* as relevant predictors and included in the linear regression analysis: age, sex, body mass index, current cigarette smoking (0, 0–15, or > 15 cigarettes/day), current alcohol consumption (yes or no), current hormone use, day of the year, long-term time trend, and temperature. We used penalized splines to account for potential nonlinearity in the relationship of day on the year [degrees of freedom (df) = 4], long-term trend (df = 3), and temperature (df = 4). The df of the penalized splines were selected *a priori*. Temperature presents U-shaped relations with several outcomes, and 4 dfs are sufficient to accommodate that. A recent simulation study suggests that about 6 dfs per year were sufficient to accommodate the long-term trend and seasonal patterns in mortality (Peng et al. 2006). We separated our seasonal and long-term trend terms and given that homocysteine is likely to have a less spiky seasonal pattern than mortality data, we used 4 dfs to account for the seasonal pattern.

The dependent variables in the models (fasting tHcy and PML tHcy) were log-transformed to improve normality and stabilize the variance. We performed regression analyses in R software version 2.2.1 (R Project for Statistical Computing, Vienna, Austria) using generalized additive models to evaluate the relation of tHcy with each air pollutant. Effects are expressed throughout the paper as percent difference in tHcy per interquartile range (IQR) difference in air pollutant concentrations.

## Results

The study included 488 (40.2%) male subjects and 725 (59.8%) female subjects between 11 and 84 years of age (mean age = 43.5 years) (Table 1). Only eight subjects were < 18 years of age. Fasting plasma tHcy ranged between 2.9 and 59.6 μmol/L, with a mean concentration of 9.0 μmol/L (95% CI, 8.8–9.2). PML tHcy ranged between 11.0 and 83.7 μmol/L, with a mean concentration of 24.2 μmol/L (95% CI, 23.8–24.6). Both fasting and PML tHcy exhibited a positive association with female sex, cigarette smoking, alcohol, and body mass index (BMI) (Table 1). Older subjects had higher fasting plasma tHcy, but PML tHcy was not significantly associated with age. To evaluate possible confounding effects, we generated linear regression models that included sex, cigarette smoking, alcohol, BMI, and age as independent variables. These models showed an independent effect of sex (*p* < 0.001 on fasting plasma tHcy; *p* < 0.001 on PML tHcy) and smoking (*p* = 0.03 on fasting plasma tHcy; *p* = 0.002 on PML tHcy), but no significant associations were found for age (*p* = 0.07 for fasting plasma tHcy; *p* = 0.19 for PML tHcy), alcohol (*p* = 0.30 for fasting plasma tHcy; *p* = 0.92 for PML tHcy), or BMI (*p* = 0.91 for fasting plasma tHcy; *p* = 0.49 for PML tHcy)

We estimated air pollution exposure on the basis of ambient measurements taken during the study period. Air pollution levels and weather variables in the study area from 1 January 1995 to 1 September 2005 are summarized in Table 2.

Table 3 presents the estimated mean differences of fasting and PML tHcy associated with an IQR increase in the average concentrations of air pollutants in the 24 hr or in the 7 days before the study. The estimates were adjusted for age, sex, body mass index, cigarette smoking, alcohol consumption, and hormone use. In addition, penalized smoothing splines were used in the models to adjust for nonlinear effects of day of the year, long-term time trend, and temperature.

PM_10_ levels did not show a significant associations with fasting and PML tHcy. The estimated increase in tHcy associated with the average PM_10_ concentrations in the 24 hr before the study was 0.4% (95% CI, −2.4 to 3.3) for fasting and 1.1% (95% CI, −1.5 to 3.7) for PML tHcy (differences were not statistically significant). Increases in 7-day PM_10_ levels were associated with nonsignificant 1.0% (95% CI, −1.5 to 3.7) and 2.0 (95% CI, −0.6 to 4.7) increases in fasting and PML tHcy, respectively. Among the gaseous pollutants, the average concentrations of O_3_ in the 24 hr before the study were significantly associated with a 6.7% (95% CI, 0.9–12.8) increase in fasting tHcy, but the association with PML tHcy was not significant (3.6%; 95% CI, −1.4 to 9.0). No association was found between the 7-day O_3_ average levels and fasting or PML tHcy. Similarly, the other gaseous pollutants were not associated with tHcy levels (Table 3).

**Figure 2:**
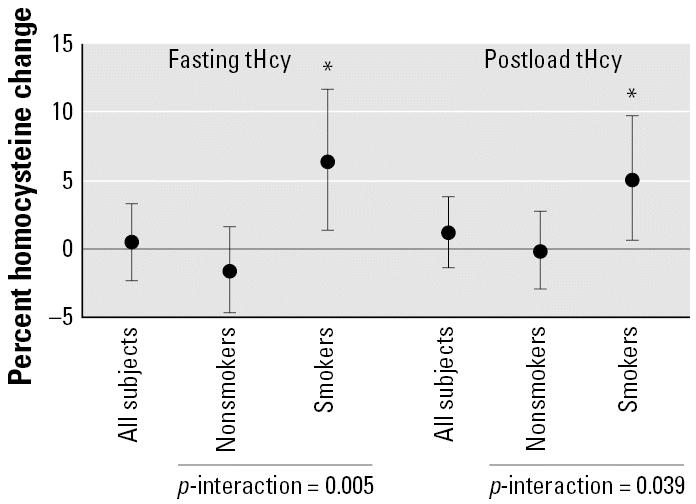
Estimates of the effect on fasting homocysteine of an IQR increase in the average concentration of PM_10_ during the 24 hr before the study, by cigarette smoking. *p < 0.05.

We then evaluated the relation between air pollution exposure, smoking, and plasma tHcy levels. Among smokers, 24-hr PM_10_ levels were associated with 6.3% (95% CI, 1.3–11.6; *p* < 0.05) and 4.9% (95% CI, 0.5–9.6; *p* < 0.05) increases in fasting and PML tHcy, respectively (Figure 2). In nonsmokers, the estimated differences were −1.7% (95% CI, −4.8 to 1.5) for fasting and −0.3 (95% CI, −3.1 to 2.6) for PML tHcy. The tests for interaction between PM_10_ and smoking were statistically significant (*p* = 0.005 for fasting tHcy; *p* = 0.039 for PML tHcy), whereas smoking in such models was not associated per se with differences in fasting (main effect = −5.9%; 95% CI −14.1 to 3.2; *p* = 0.20) or PML tHcy (main effect = −0.6%; 95% CI −8.5 to 8.0; *p* = 0.89). The association between the PM_10_ levels in the 7 days before the study and tHcy showed the same pattern. For smokers, 7-day PM_10_ was associated with a nonsignificant 3.3% (95% CI, −1.5 to 8.4) increase in fasting tHcy and a significant 5.2% (95% CI, 0.8 to 9.8; *p* < 0.05) increase in PML tHcy. Although no significant increase in tHcy was found among non-smokers (0.1%, 95% CI, −3.2 to 3.3 for fasting tHcy; 0.7%, 95% CI, −2.2 to 3.7 for PML tHcy), the tests for interaction between average 7-day PM_10_ and smoking were not statistically significant (*p* = 0.23 for fasting tHcy; *p* = 0.07 for PML tHcy).

Our main results for PM_10_ exposure reported above were based on analysis that included both measured PM_10_ and predicted PM_10_ values that were used for the earlier time periods in which TSP rather than PM_10_ data were available (see “Materials and Methods”). The results based only on measured PM_10_ confirmed the presence of a statistical interaction between PM_10_ and smoking. Among smokers, measured 24-hr PM_10_ levels were associated with 6.2% (95% CI, 0.0–12.7; *p* < 0.05) and 6.0% (95% CI, 0.5–11.8; *p* < 0.05) increases in fasting and PML tHcy, respectively. In non-smokers, the estimated differences were −1.6% (95% CI, −5.5 to 2.5) for fasting and −0.1 (95% CI, −3.6 to 3.5) for PML tHcy. Again, the tests for interaction between measured PM_10_ and smoking were statistically significant (*p* = 0.026 for fasting tHcy; *p* = 0.048 for PML tHcy). As for the main analysis based on both measured and predicted PM_10_ levels, measured mean PM_10_ levels in the 7 days before the study were associated with higher tHcy in smokers but not in nonsmokers, whereas the interaction terms were again not statistically significant (data not shown).

Gaseous pollutants (CO, NO_2_, SO_2_, and O_3_) showed no significant interaction with cigarette smoking in association with the levels of fasting (*p* > 0.26) and PML tHcy (*p* > 0.43). In addition, no significant interaction of the air pollutants evaluated with age, sex, alcohol use, overweight (BMI > 25), and obesity (BMI > 30).

## Discussion

In this study conducted on a large sample of subjects from Lombardia, Italy, air pollution levels measured in the week preceding the study did not show overall consistent associations with fasting and PML tHcy. However, we found that PM_10_ interacted with cigarette smoking in determining increased tHcy levels. PM_10_ levels, particularly those in the 24 hr before the study, were associated with increased fasting and PML tHcy in smokers but not in nonsmokers.

Tobacco smoking is one of the strongest risk factors for cardiovascular disease. Previous studies indicate that smoking is independently associated with increased tHcy levels in patients with coronary artery disease, ischemic stroke, and diabetes as well as in the general population (De Bree et al. 2002; Nygard et al. 1995; Targher et al. 2000). Several mechanisms have been suggested to account for the smoking-related increase in tHcy, including changes in plasma thiol redox status, possibly because of a higher formation of reactive oxygen species; inactivation of the enzymes of homocysteine remethylation, such as methionine synthase; reduced intake of nutrients and vitamins; and lower levels of plasma folate, vitamin B12, and plasma pyridoxal 5-phosphate (De Bree et al. 2002).

Oxidative stress and endothelial dysfunction, which are enhanced in subjects with hyperhomocysteinemia (De Bree et al. 2002), have been associated with both cigarette smoking (Targher et al. 2000) and exposure to air particles (Brook et al. 2004; Utell et al. 2002). It has been suggested that noncompensated oxidative stress may contribute to the increase in plasma homocysteine concentrations by subtracting from the synthesis of homocysteine methyl group donors that are used to compensate cell oxidative damage (Gori et al. 2005). It is possible that the inflammatory status induced by cigarette smoking produces an increased demand for methyl group donors that may be exacerbated by air particle exposure. Thus, smoking may amplify the effects of PM_10_ on homocysteine metabolisms and produce the association that we observed among smokers. The results of previous investigations have shown that people with congestive heart failure, conduction disorders, myocardial infarction, chronic obstructive pulmonary disorder, and diabetes are at greater risk of adverse events associated with air pollution in general and specifically with particulate matter (Bateson and Schwartz 2004). Smoking is strongly associated with most of these conditions and may represent the underlying modifier determining the stronger air pollution effects observed in those high-risk groups.

Our estimates for the association of PM_10_ levels with increased tHcy among smokers indicate that an IQR difference in PM_10_ average concentration in the preceding 24 hr is associated with a 6.3% increase in fasting tHcy and a 4.9% increase in PML tHcy (Figure 2). The clinical significance of the PM_10_-related increases in tHcy in our study is uncertain and should also be interpreted in the light of the results of recent large multicenter clinical trials on homocysteine-lowering treatment that failed to demonstrate a reduction in major cardiovascular events in high-risk subjects with previous acute myocardial infarction (Lonn et al. 2006) or preexisting cardiovascular disease or diabetes (Bonaa et al. 2006). These results may suggest that differences in tHcy such as those observed in our study may represent indicators of increased cardiovascular risk, rather than causal determinants of cardiovascular disease.

In our previous work on this same population (Baccarelli et al. 2006), we found that PM_10_ exposure was associated with shortened prothrombin time, suggesting the presence of exposure-related hypercoagulability in the same subjects of this present study. However, cigarette smoking did not modify the association of prothrombin time with PM_10_ levels, thus indicating that the interaction with smoking could be operating through mechanisms that are specific to the tHcy pathway.

In addition, we found a positive association between O_3_ levels measured in the 24 hr before the study and fasting tHcy. Short-term exposure to O_3_, one of the most potent single oxidants in the ambient air pollutant mixture, has been associated with decreased heart rate variability (Schwartz et al. 2005), ventricular arrhythmia (Rich et al. 2005), ischemic heart disease (Lee et al. 2003), and cardiovascular mortality (Gryparis et al. 2004). O_3_ has been shown to produce inflammatory reactions in the respiratory tract (Aris et al. 1993; Balmes et al. 1996), as well as systemic inflammation and procoagulant status (Hermans et al. 2005). However, our results on the association of O_3_ with tHcy did not show a consistent pattern when we evaluated postload tHcy levels and no significant associations were found when the 7-day average of O_3_ levels was used in the analyses.

A limitation of our study is that we used ambient air pollution as a surrogate for personal exposure, which may have resulted in measurement error. Such measurement error would generally tend to bias estimates toward the null (Samet et al. 2000a) and may have contributed to the overall lack of association in our study. However, a recent study comparing personal exposures to site monitoring in Boston, Massachusetts, reported that monitor readings and personal exposure are highly correlated (Sarnat et al. 2005). Moreover, it has been suggested that the consequence of using ambient measures to estimate exposure is likely to be only a modest underestimation of pollution effects (Zeger et al. 2000). Our study was based on readings of hourly air pollution data from 53 different monitoring sites throughout Lombardia Region that were selected on the basis of their capability to represent local background air pollution, as determined by correlation with random *in situ* measurements in the adjacent territory. The analysis was based on the average concentrations in nine different pollution areas, to which study subjects were assigned based on their residence. The nine areas showed spatially homogenous pollution patterns, as determined by the high correlation of the measures from the monitoring stations in the same area, as well as of measurements performed at different within-area locations during the study period. In addition, we considered in the analysis several potential confounding factors that may have influenced tHcy. Age, sex, body mass index, alcohol consumption, hormone use, day of the study, and temperature did not confound the association between air pollution and homocysteine. Therefore, chances that the observed associations reflected bias due to confounding factors are minimized.

In conclusion, our study demonstrated no consistent association of air pollution levels with fasting and postmethionine load tHcy when all subjects where considered. However, we found increased tHcy levels in association with higher concentrations of ambient PM_10_ among smokers, suggesting that cigarette smoking and air pollution may interact in increasing plasma tHcy levels in healthy subjects.

## Figures and Tables

**Table 1:** Fasting and postmethionine-load total homocysteine levels, by study subjects’ characteristics.

		Fasting total homocysteine (μmol/l)		Postmethionine-load total homocysteine (μmol/L)	
	No. of subjects	Mean (95% CI)^a^	*p*-Value^b^	Mean (95% CI)^a^	*p*-Value^b^
All subjects	1,213	9.0 (8.8–9.2)	—	24.2 (23.8–24.6)	—
Age (years)					
< 35	370	8.7 (8.4–9.1)		23.6 (22.8–24.3)	
35–55	270	9.0 (8.6–9.4)		24.7 (23.8–25.7)	
45–55	312	9.2 (8.8–9.5)		24.1 (23.4–24.8)	
55–65	209	9.1 (8.7–9.5)		24.9 (23.9–25.9)	
> 65	52	9.6 (8.9–10.4)	0.04	24.1 (22.5–25.8)	0.13
Sex					
Male	488	10.4 (10.1–10.8)		25.8 (25.1–26.5)	
Female	725	8.1 (8.0–8.3)	< 0.001	23.1 (22.6–23.6)	< 0.001
Smoking (cigarettes/day)					
No	870	8.9 (8.7–9.1)		23.8 (23.4–24.3)	
1–15	205	9.1 (8.6–9.6)		24.7 (23.6–25.8)	
> 15	138	9.6 (9.1–10.2)	0.02	25.8 (24.5–27.3)	0.005
Alcohol					
No	559	8.5 (8.3–8.8)		23.7 (23.1–24.3)	
Yes	645	9.4 (9.2–9.7)	< 0.001	24.6 (24.0–25.1)	0.04
Coffee (cups/day)					
No	158	8.8 (8.3–9.2)		23.4 (22.3–24.6)	
1	218	8.9 (8.5–9.4)		24.3 (23.3–25.4)	
2	336	9.1 (8.8–9.5)		24.3 (23.6–25.1)	
3	262	8.8 (8.4–9.1)		23.9 (23.1–24.8)	
> 3	239	9.3 (8.9–9.7)	0.34	24.7 (23.7–25.6)	0.27
Body mass index (kg/m^2^)					
< 21	285	8.4 (8.1–8.8)		23.3 (22.4–24.1)	
21–23.5	326	8.9 (8.5–9.2)		24.3 (23.5–25.1)	
23.5–26	295	9.0 (8.7–9.4)		23.9 (23.1–24.7)	
> 26	301	9.7 (9.3–10.1)	< 0.001	25.3 (24.4–26.2)	0.007
Hormone use^c^					
No	527	8.1 (7.9–8.3)		22.9 (22.3–23.4)	
Yes	167	8.1 (7.8–8.4)	0.84	23.5 (22.5–24.6)	0.25

a Geometric means are reported to account for lognormal tHcy distributions.

b *p*-Value for trend across multiple categories or Student’s t-test for differences between categories of binomial variables.

c Women who used oral contraceptives or hormone replacement therapy at the time of blood sampling.

**Table 2:** Air pollution profile and weather variables in Lombardia Region, Italy, from 1 January 1995 to 1 September 2005.^a^

		Percentile			
	No.^b^	25th	Median	75th	Maximum
Air pollutants					
PM_10_ (μg/m^3^)	776,318	20.1	34.1	52.6	390.0
CO (ppm)	822,034	0.50	0.85	1.50	20.59
NO_2_ (ppb)	821,363	13.6	22.7	33.7	194.2
SO_2_ (μg/m^3^)	822,180	3.2	6.3	11.8	253.3
O_3_ (ppb)	810,509	7.0	18.3	35.1	202.3
Weather variables					
Temperature (°C)	−17.3	6.0	12.9	19.8	41.2
Barometric pressure (mmHg)	850.0	982.4	996.3	1005.0	1050.0
Relative humidity (%)	0.0	57.6	76.1	91.8	100.0

a Average of hourly measurements from multiple monitors located in each of the nine pollution areas. Concentrations were missing for the earlier periods of the study in some of the study areas (total possible hours, *n* = 841,536).

b For weather variables, minimum.

**Table 3:** Estimates of the effect of IQR increase in air pollutants on fasting and postmethionine-load homocysteine.

	24-hr moving average		7-day moving average	
	Pollutant IQR	Homocysteine difference^a^ Percent (95% CI)	Pollutant IQR	Homocysteine difference^a^ Percent (95% CI)
Homocysteine, fasting^b^				
PM_10_	32.5	0.4 (−2.4 to 3.3)	25.7	1.0 (−1.9 to 3.9)
CO	1.3	−0.8 (−3.7 to 2.2)	1.3	−2.4 (−5.6 to 0.8)
NO_2_	16.8	0.2 (−2.4 to 3.0)	16.3	−1.8 (−4.9 to 1.4)
SO_2_	17.1	0.1 (−4.1 to 4.4)	11.2	0.1 (−2.8 to 3.1)
O_3_	21.4	6.7 (0.9 to 12.8)*	21.6	4.5 (−1.9 to 11.3)
Homocysteine, postmethionine-load^b^				
PM_10_	32.5	1.1 (−1.5 to 3.7)	25.7	2.0 (−0.6 to 4.7)
CO	1.3	−0.4 (−3.0 to 2.3)	1.3	0.5 (−2.4 to 3.5)
NO_2_	16.8	0.0 (−2.4 to 2.4)	16.3	0.4 (−2.4 to 3.4)
SO_2_	17.1	1.7 (−2.1 to 5.6)	11.2	1.7 (−1.0 to 4.4)
O_3_	21.4	3.6 (−1.4 to 9.0)	21.6	−0.7 (−6.1 to 5.1)

a Percent difference in plasma homocysteine per IQR increase in air pollutant, adjusted for age, sex, BMI, cigarette smoking, alcohol consumption, oral contraceptives, and penalized smoothing splines for day of the year (df = 4), long-term time trend (df = 3), and temperature (df = 4).

b Total plasma homocysteine measured by high-performance liquid chromatography after overnight fasting and 4 hr after an oral methionine loading (3.8 g/m2 of body surface area).

* *p* < 0.05 for the association with pollutant levels.
